# Generation of *six4* -nlsRFP: a red somatic gonadal nuclear marker for live imaging *Drosophila* gonadogenesis

**DOI:** 10.17912/micropub.biology.001652

**Published:** 2025-07-01

**Authors:** Everette Rhymer, Lauren Anllo

**Affiliations:** 1 Biology, East Carolina University

## Abstract

Intrinsically fluorescent tissue labels are valuable to live image cell behaviors during development. Fluorescent markers of *Drosophila* gonadal cells have elucidated dynamic cellular interactions that shaped our conception of cell biology. Many green fluorescent protein (GFP) reagents are available to visualize cytoskeletal dynamics, extracellular matrix, and cell adhesion proteins. To facilitate use of these reagents while studying somatic gonadal development, we generated a somatic gonadal precursor (SGP) intrinsic red fluorescent nuclear marker. This tool can track SGP movement live, facilitating the study of somatic cell interactions required for gonadogenesis.

**Figure 1. Generation of six4 nlsRFP somatic gonadal reporter:**
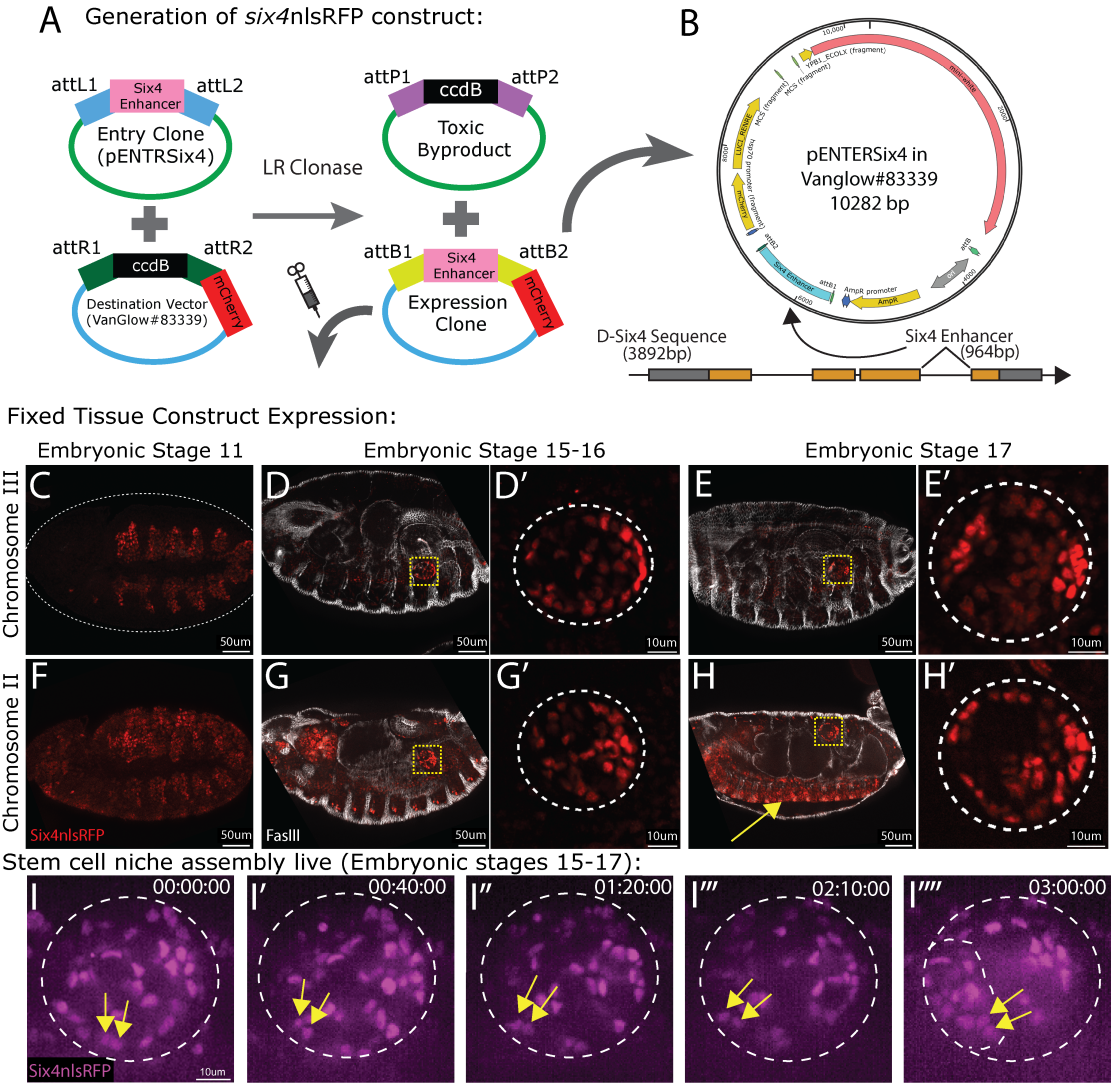
**(A)** LR clonase reaction used to insert the *Six4* enhancer sequence upstream of the nuclear red fluorescent protein sequence, mCherry:Renilla(nls), in the Vanglow#83339 plasmid. Entry clone and toxic byproduct plasmids are Kanamycin resistant (KanR), and destination vector and expression clone are Ampicillin resistant (AmpR). The construct injected is labeled as expression clone. **(B)** Sequence map of the plasmid generated to develop the *six4* nlsRFP reporter. The *Six4* enhancer noted on the map is the third intron of the D- *Six4* gene sequence, diagrammed below the plasmid (exons, orange; UTRs, gray; introns, horizontal line). **(C-H)** Whole embryo, fixed tissue immunostaining for RFP to show *six4* nlsRFP reporter expression for the construct injected on chromosomes III **(C-E)** and II **(F-H)** at embryonic stages 11 **(C,F)** , 15-16 **(D,G)** , and 17 **(E,H)** with the gonad outlined by yellow boxes. Expression of *Six4* in the ventral nervous system (H, yellow arrow). Prime panels show high magnification of gonads in corresponding panels, outlined with dotted white line. RFP, red; Fas3, white. ( **I-M** ) Live imaging of stem cell niche assembly during embryonic stages 15-17 using the *six4* nlsRFP reporter (magenta) to show SGP nuclei. Two individual niche cells over time (I-I'''', yellow arrows). Gonad outlined in white. Niche within gonad in I'''' also outlined in white. Time indicated in hh:mm:ss.

## Description

**Introduction**

Fluorescent proteins are commonly used to label subcellular structures during live imaging, which has enabled a way to study cell biological processes *in vivo* during development (Chudakov et al., 2005, 2010; Day & Davidson, 2009). A prominent model that has defined many foundational developmental and reproductive processes is the *Drosophila* embryonic gonad (Heemskerk & DiNardo, 1994; Kunwar et al., 2006; Le Bras & Van Doren, 2006; Santos & Lehmann, 2004; Sheng et al., 2009; Tran et al., 2000; Yamashita Yukiko et al., 2003). At embryonic stage 15, the gonad is a spherical arrangement of germ cells (GCs) encysted by somatic gonadal precursor cells (SGPs). The development of intrinsically fluorescent protein labels has enabled live imaging to describe coalescence of these gonadal cell types, and testis stem cell niche morphogenesis (Anllo et al., 2019; Clark et al., 2006; Lin et al., 2020; Sano et al., 2012; Warder et al., 2024). Libraries of intrinsic labels to visualize cytoskeletal regulators, adhesion proteins, and extracellular matrix components often employ green fluorescent protein (GFP) (Nagarkar-Jaiswal et al., 2015). To use these libraries while simultaneously visualizing formation of somatic gonadal structures including the niche, the construction of red fluorescent SGP labels is crucial. The field previously lacked a tool to intrinsically label SGPs with a red fluorophore. This limitation prevented red fluorescent labeling of SGPs while simultaneously using Gal4/UAS drivers in other gonadal tissues. Here, we used molecular cloning to develop a *six4* nlsRFP genetic fly line that intrinsically labels SGP nuclei with an mCherry RFP. This line enables the tracking of somatic gonadal cell migratory paths during live imaging, while simultaneously labeling other proteins of interest in separate fluorescent channels. We show that our *six4* nlsRFP lines can be used to visualize SGPs in both fixed tissue immunostaining and live imaging experiments at various stages of embryonic gonadogenesis.

**Results**

*Transgenic expression of six4nls RFP in fixed tissue:*

Embryos expressing the *six4nls* RFP sequence were fixed and immunostained against RFP to visualize the SGP reporter. The adhesion protein Fasciclin III was also immunostained to highlight specific structures in the embryo, including the epidermis and visceral muscle. Embryos with *six4* nlsRFP on chromosome 3 (see Methods) showed nuclear expression in a subset of mesodermal cells during mid-embryogenesis ( Fig. 1C ), and more restricted expression later in SGPs during gonad coalescence and formation of the spermatogonial niche ( Fig. 1C- 1E'). Interestingly, embryos with the *six4* nlsRFP sequence inserted at the docking site on chromosome 2 showed additional RFP expression in the nervous system, along with expression in early mesoderm and later somatic gonadal cells ( Fig. 1F- H'). Thus, while both insertions allow for clear delineation of SGPs, the positional insertion on Chromosome III enables more specific SGP visualization.

*Transgenic expression of six4nls RFP in live imaging:*

Once expression was confirmed in fixed tissue experiments, we used our chromosome 3 inserted *six4* nlsRFP-expressing embryos to live image assembly of the spermatogonial stem cell niche. The spermatogonial niche derives from a subset of SGPs that cluster together at the anterior of the male gonad during Stages 15-17 of embryogenesis (Le Bras & Van Doren, 2006; Anllo et al., 2019). Endogenous expression of the construct was sufficient to visualize the gonad and mount embryos for live imaging at embryonic stage 15 ( Fig. 1I ). After 3 hours of live imaging, at embryonic stage 17, we observed a niche assembled at the anterior ( Fig. 1I ''''). The *six4* nlsRFP construct allowed visualization and tracking of individual niche cells migrating to the anterior ( Fig. 1I- 1I'''', yellow arrows). We show here that the *six4* nlsRFP construct can be used to trace the migratory paths and dynamic movements of individual SGPs live during niche assembly.

**Discussion**

During embryogenesis, the gonad undergoes rapid morphological changes (Clark et al., 2006; Jenkins et al., 2003; Sano et al., 2012). Live imaging the late embryonic *Drosophila* testis has revealed cell migratory paths and cytoskeletal dynamics that could not have been predicted from fixed tissue image analysis (Anllo et al., 2019; Anllo & DiNardo, 2022; Warder et al., 2024). To investigate these behaviors, precise intrinsic labeling of individual cells is required along with separate visualization of extracellular matrix interactors, cell-cell adhesive proteins, and cytoskeletal dynamics. Here, we generated a red fluorescent marker, *six4* nlsRFP, to intrinsically mark somatic gonadal precursor (SGP) nuclei, enabling tracking of individual cells while employing GFP-tagged libraries to separately label cell interactions and behaviors. The *six4* nlsRFP reporter does not require use of Gal4/UAS induction, thus saving Gal4/UAS for other simultaneous manipulations in SGPs, germ cells, or other extrinsic tissues.

Despite lower quantum yield of red fluorescent proteins compared to green fluorescent variants (Wall et al., 2015), *six4* nlsRFP has high intrinsic fluorescence for visualization with a stereofluorescent dissecting microscope. This feature enables precise orientation of embryos mounted for live imaging with the gonad positioned directly adjacent to the coverslip. One copy of the *six4* nlsRFP construct provides sufficient fluorescence despite the internal depth of the gonad more than 50 microns from the epidermis. Additionally, chromosomes with the *six4* nlsRFP construct are homozygous viable, presenting the opportunity for increased brightness with two construct copies.

The chromosome 3 attP2 insertion of *six4* nlsRFP shows more restricted expression than the chromosome 2 attP1 insertion, which has ectopic expression in the nervous system. Positional effects have been documented for attP sites that vary across tissues (Markstein et al., 2008), and strong position effects were found for attP1 in the adult nervous system when an enhancer trap vector was inserted (Peiffer et al., 2010). These position effects might explain the additional RFP expression detected in the embryonic nervous system with the attP1 insertion.

Individual SGPs can be tracked over time using the *six4* nlsRFP label. During late-stage gonadogenesis, SGPs encyst germ cells with long cellular extensions that contact one another in dense cellular arrangements (Clark et al., 2006; Sano et al., 2012). This arrangement poses a challenge for tracking individual cell positions with cell cortex labels. The nuclear fluorescence with *six4* nlsRFP overcomes this limitation. Our future work will employ this tool to track individual SGPs while using separate GFP fluorophores to measure their dynamic F-actin nucleation patterns during interactions with the germline, extracellular matrix, and other SGPs.

This tool facilitates study of cell behaviors that are not detectable in individual cells over time in fixed immunostaining experiments. Overall, this reagent enhances our ability to investigate dynamics in the developing somatic *Drosophila* gonadal cells, which has implications for advancing studies of stem cell-niche biology and organogenesis.

## Methods

*Generation of six4nlsRFP expressing flies:*

To generate a *six4* nlsRFP DNA sequence, we used two genomic plasmids, the Gateway vector pENTR (ThermoFisher Scientific) and Vanglow#83339 (AddGene, Janssens et al., 2017). We used Gateway cloning to insert the *Six4* enhancer sequence into pENTR, flanked by 2 attL sites ( Fig. 1A ). Oligonucleotides previously described were used to amplify the *Six4* enhancer encoded in its third intronic region (Clark et al., 2006). The Vanglow#83339 plasmid contained a ccdB sequence, encoding a cell death casette, flanked by 2 attR sites and followed by a red fluorescent protein (RFP) sequence, mCherry, with a nuclear localization signal. pENTR and contained a kanamycin-resistance gene (KanR), while Vanglow#83339 encoded ampicillin-resistance (AmpR) sequence, allowing for selective growth when transformed in bacteria. Plasmids were amplified using bacterial transformation and validated using restriction enzyme digestion and DNA sequencing (Plasmidsaurus).

An LR clonase reaction was performed to induce recombination at the attL and attR sites between these plasmids (ThermoFisher Scientific). This reaction placed the *Six4* enhancer sequence upstream of the mCherry RFP sequence in VanGlow#83339 ( Fig. 1B ). Plasmids were then amplified using bacterial transformation and grown selectively on agar plates containing 100 ug/uL ampicillin. Bacterial cultures containing *six4nlsRFP* were purified and sequenced (Plasmidsaurus), which validated that the LR clonase reaction had worked successfully. The sequence was then injected into flies containing an attP1 docking site on chromosome 2R (55C4) (BDSC#8621), and fly lines containing an attP2 docking site on chromosome 3L (68A4) (BDSC#8622) through PhiC31 integrase-mediated transformation (Bateman et al., 2006). BestGene was used for injections.

*Immunostaining and imaging:*

Embryos were collected on grape agar plates overnight, then dechorionated using 50% bleach for approximately 2 minutes. Embryos were transferred to a glass screw cap vial with equal parts heptane and 4% paraformaldehyde (PFA) to fix for 20 minutes on a rocking nutator. PFA was then aspirated and replaced with an equal amount of 100% methanol, and the glass vial was vigorously shaken for 1 minute to remove the vitelline membrane. Two washes of 100% methanol were performed to wash away excess PFA and heptane. Embryos were then washed in 50/50 methanol/PBS to rehydrate, then placed in 0.1% phosphate-buffered saline with Triton (PBST) prior to blocking. Tissue was blocked using 4% normal donkey serum (NDS). Primary antibody staining was performed overnight at 4C, and all secondary antibodies were used at 3.75ug/ml (Alexa 488, Cy3, Alexa 647; Molecular Probes; Jackson Immunoresearch) for 2 hours at room temperature. Hoechst 33342 (Sigma) was used at 0.2ug/ml for 5 minutes to stain DNA. We used rabbit antibody against RFP at 1:500 (Abcam 62341) and mouse antibody against Fasciclin III at 1:20 (DSHB 7G10). Fixed embryos were imaged using a Zeiss AxioImager.Z1 with an ApoTome.2, X-cite Xylis fluorescent light source, and Axiocam 705 mono camera, using a 20x 0.8 N.A. lens, or a 40x 1.2 N.A. lens.

*in vivo live imaging:*

Embryos were prepared for live imaging as described previously by Ong and colleagues (Ong et al., 2019). Embryos were collected overnight (~16hrs) at 25C on a grape agar plate and dechorionated the next morning in a nytex basket using 50% bleach. Embryos were then selected for embryonic stage 15 using gut morphology to determine age according to Campos Ortega and Hartenstein (Campos-Ortega & Hartenstein, 1997). Embryos were mounted dorsolaterally to a slide using double-sided tape dissolved in heptane to create an adhesive solution and then covered in halocarbon 27 oil. Two 18x18mm coverslips were glued to the slide on both sides of the embryos to create a bridge. A 22x30mm coverslip was then glued to the bridge coverslips to allow space for the embryos. Slides were imaged using an Olympus IX83 microscope with a Crest X-Light V3 spinning disk, using a 60x oil immersion objective.

## Reagents

**Table:** 

**STRAIN**	**GENOTYPE**	**AVAILABLE FROM**
*Drosophila melanogaster*	y[1] w[67c23]; P{y[+t7.7]=CaryP}attP1	Bloomington *Drosophila* Stock Center #8621
*Drosophila melanogaster*	y[1] w[67c23]; P{y[+t7.7]=CaryP}attP2	Bloomington *Drosophila* Stock Center #8622
*E. coli* One Shot Top 10 competent cells		Invitrogen, Catalog # C404006
**PLASMID**	**GENOTYPE**	**DESCRIPTION**
Vanglow#83339		mCherry::Renilla(nls) upstream of ccdB flanked by two AttR cloning sites. Available at Addgene. Catalog # 83339.
pENTR/D-Topo entry vector		Invitrogen, Catalog # K240020.
*Six4* enhancer oligonucleotides	CAC CCA GCA AAG ACC GTG AGT TG	(Clark et al., 2006) D- *Six4* third intron, forward, EcooR1 site
*Six4* enhancer oligonucleotides	GTT GGA TCC ATT GCC ATC CAG TTG	(Clark et al., 2006) D- *Six4* third intron, reverse, BamH1 site
**ANTIBODY**	**SOURCE**	**IDENTIFIER**
Rabbit polyclonal anti RFP	Abcam	ab62341; RRID:AB_945213
Mouse monoclonal anti FasciclinIII	Developmental Studies Hybridoma Bank	DSHB-7G10 RRID:AB_528238
Alexafluor Secondary Antibodies (Cy5 conjugated) (minimally cross reactive IgG generated in donkey)	Jackson ImmunoResearch laboratories	Catalog # 715-175-151
Cy3 Affinipure Secondary Antibodies	Jackson ImmunoResearch laboratories	Catalog # 711-165-152
**CHEMICAL REAGENT**	**SOURCE**	**IDENTIFIER**
Gateway LR Clonase II Enzyme mix	Thermofisher Scientific	Catalog # 11791020
Ampicillin	Thermofisher Scientific	Catalog # 11593027
Kanamycin	Thermofisher Scientific	Catalog # 11815032
**EQUIPMENT**	**SOURCE**	**IDENTIFIER**
Leica M165FC	Leica	N/A
mCherry Filter set ET560/40x; ET630/75m	Leica 10450195	N/A
Achromat 1.6x objective	Leica 10450163	N/A
Olympus IX83 microscope	Olympus/Evident	N/A
Crest X-Light V3 spinning disk confocal microscope	89 North	N/A
Silicone oil objective 60x, 1.3 NA	Evident 1-U2B7122	N/A
Zeiss AxioImager.Z1 microscope equipped with ApoTome.2 and X-Cite Xylis LED illuminator	Zeiss	N/A
C-Apo 40x water objective 1.2 NA	Zeiss	N/A
Plan-Apo 20x objective 0.8 NA	Zeiss	N/A
